# Supplementary material for: PET/CT based cross-modal deep learning signature to predict occult nodal metastasis in lung cancer
Source: Nat Commun. 2023 Nov 18;14:7513. doi: 10.1038/s41467-023-42811-4 (PMC10657428; doi:10.1038/s41467-023-42811-4)
Supplement: Supplementary file 1 — Supplementary Information [file 41467_2023_42811_MOESM1_ESM.pdf]

List of Supplementary Material

Part I. Supplementary Methods

Supplementary Method 1. Inclusion and exclusion criteria
Supplementary Method 2. Follow-up protocol
Supplementary Method 3. Parameters of PET/CT scanners
Supplementary Method 4. Tumor annotations
Supplementary Method 5. Data pre-processing process
Supplementary Method 6. Neural network training process
Supplementary Method 7. Selection for deep learning architecture, fusion strategy, and image augmentation
Supplementary Method 8. Adjustments among multiple facilities
Supplementary Method 9. Benchmarking
Supplementary Method 10. ONM scores of patients receiving limited nodal dissection
Supplementary Method 11. Gene set enrichment analysis
Supplementary Method 12. Single sample gene set enrichment analysis

Part II. Supplementary Tables

Supplementary Table 1. Logistic analyses of N1 and N2 metastasis before the incorporation of DELNS for patients in the validation set, external cohort and prospective cohort
Supplementary Table 2. Logistic analyses of N1 and N2 metastasis after the incorporation of DLNMS for patients in the training set
Supplementary Table 3. Logistic analyses of N1 and N2 metastasis after the incorporation of DLNMS for patients in the validation set, external cohort and prospective cohort
Supplementary Table 4. Integrated discrimination improvement and net reclassification index of DLNMS for occult N1 and N1 prediction
Supplementary Table 5. Baseline characteristics of patients receiving nodal biopsy
Supplementary Table 6. Baseline characteristics of clinical stage I patients receiving limited lymph node dissection

Part III. Supplementary Figures

Supplementary Fig. 1. Scatter graphs illustrating the DLNMS score distributions in the training set, validation set, external cohort and prospective cohort
Supplementary Fig. 2. Confusion matrixes of the DLNMS to identify occult nodal metastasis in the training set, validation set, external cohort and prospective cohort
Supplementary Fig. 3. Scatter graphs illustrating the DLNMS correct cases falsely predicted by the (A & B) PET and (C & D) CT models in the validation set, external cohort and prospective cohort
Supplementary Fig. 4. Calibration curves of the DLNMS to identify occult N1 and N2 metastasis
Supplementary Fig. 5. Decision curves of the DLNMS to identify occult nodal metastasis in the training set, validation set, external cohort and prospective cohort

Supplementary Fig. 6. Prognosis of patients with non-small cell lung cancer grouped by different risk scores in the validation set and external cohort
Supplementary Fig. 7. Flow chart illustrating patient selection
Supplementary Fig. 8. Diagram illustrating the structure of DLNMS

Part IV. References

Supplementary Method 1. Inclusion and exclusion criteria

In the internal cohort, consecutive patients receiving curative resection for clinical stage N0 non-small cell lung cancer (NSCLC) from January 2018 to December 2019 at Shanghai Pulmonary Hospital were retrospectively reviewed. Criteria for inclusion were as follows: (a) patients receiving curative surgery for primary NSCLC; (b) the maximum short-axis diameter of N1 and N2 lymph nodes less than 1 cm on CT scan; (c) the maximum standardized uptake value (SUVmax) of N1 and N2 lymph nodes less than 2.5. Criteria for exclusion included (a) multiple lung lesions; (b) poor quality of positron emission tomography/computed tomography (PET/CT) images; (c) patient not receiving systematic nodal dissection (SND); (d) patient receiving neoadjuvant therapy; and (e) lost to follow-up.

In the external cohort, we retrospectively included patients receiving curative resection for clinical stage N0 NSCLC from January 2018 to December 2019 at The First Hospital of Nanchang University, Affiliated Hospital of Zunyi Medical College and Ningbo HwaMei Hospital. Criteria for inclusion were as follows: (a) patients receiving curative surgery for primary NSCLC; (b) the maximum short-axis diameter of N1 and N2 nodes less than 1 cm on CT scan; (c) the SUVmax of N1 and N2 lymph nodes less than 2.5. Criteria for exclusion included (a) multiple lung lesions; (b) poor quality of PET/CT images; (c) patient not receiving SND; (d) patient receiving neoadjuvant therapy; and (e) lost to follow-up.

In the prospective cohort, participants receiving curative resection for clinical stage N0 NSCLC from January 2022 to June 2022 at Shanghai Pulmonary Hospital, The First Affiliated Hospital of Nanchang University, Affiliated Hospital of Zunyi Medical College and Ningbo HwaMei Hospital were enrolled. Criteria for enrollment were as follows: (a) participants scheduled for surgery for radiological finding of pulmonary lesions from the preoperative thin-section CT scans; (b) the maximum short-axis diameter of N1 and N2 lymph nodes less than 1 cm on CT scan; (c) the SUVmax of N1 and N2 lymph nodes less than 2.5; (d) pathological confirmation of primary NSCLC; (e) age ranging from 20-75 years; and (f) obtained written informed consent. Criteria for exclusion included (a) multiple lung lesions; (b) poor quality of PET/CT images; (c) participants with incomplete clinical information; (d) participants not receiving SND and (e) participants receiving neoadjuvant therapy.

In the biopsy cohort, patients receiving nodal biopsy for clinical stage N0 NSCLC from January 2020 to December 2021 at Shanghai Pulmonary Hospital, The First Affiliated Hospital of Nanchang University, Affiliated Hospital of Zunyi Medical College and Ningbo HwaMei Hospital were retrospectively reviewed. Criteria for inclusion were as follows: (a) patients receiving endobronchial ultrasound transbronchial needle aspirations for primary NSCLC; (b) the maximum short-axis diameter of N1 and N2 lymph nodes less than 1 cm on CT scan; and (c) the SUVmax of N1 and N2 lymph nodes less than 2.5. Patients with multiple lung lesions were excluded.

Supplementary Method 2. Follow-up protocol

Follow up was conducted at 3, 6, 12 months within the first postoperative year and then at one-year interval. Chest CT and abdominal ultrasound were routinely implemented. MRI scan for cerebrum and bone were adopted to exclude distant metastasis. The PET scan or/and endobronchial ultrasound-guided transbronchial needle aspiration were recommended when recurrence was suspected. Survival data were acquired from the outpatient visit and telephone follow-up. Overall survival was estimated as the duration since the day of surgery until the day of death or last follow-up visit. Recurrence-free survival was defined as the time elapsed between the date of surgery and the date of progress or death or last follow-up visit. All patients in this study completed the follow-up survey up to 2022 December.

Supplementary Method 3. Parameters of PET/CT scanners

PET/CT scans were conducted on a Siemens Biograph 64 (Erlangen, Germany), General Electric Discovery 710 (Boston, America) and General Electric Discovery STE (Boston, America). Patients were required to fast for at least 6 h prior to imaging, and serum glucose levels were kept lower than 7.4 mmol/l. Images were captured ∼60 min after intravenous administration of 3.7–6.7 MBq of 18F-FDG per kilogram of body weight. PET images were acquired for 2.5 min per bed position. All PET images were converted into SUV units by normalizing the activity concentration to the dosage of 18F-FDG injected and the patient body weight after decay correction. SUVmax was calculated as decay-corrected maximum activity concentration in the lesion.

Supplementary Method 4. Tumor annotations

PET/CT images within 2 weeks before treatments were extracted and imported into the 3D slicer software (version 4.8.0, Brigham and Women's Hospital). The region of interest (ROI) on PET and CT images was annotated with a bounding box including the primary tumor by a junior chest radiologist (T.W with 5 years of experiences) and confirmed by an expert chest radiologist (J.S with 25 years of experiences). Each annotated ROI was labeled with the corresponding occult nodal metastasis status. All reviewers were blind for clinical information in the process of tumor annotations.

Supplementary Method 5. Data pre-processing process

The data pre-processing process was divided into ten steps [1-7]: (1) Resampled each voxel in the original images to 0.6*0.6*0.6 mm3 in the spatial dimension; (2) ROI of the lesion was randomly rotated -180~180 around its center; (3) Extracted the 3D ROI with a ROI size of 150*150*150 voxels; (4) Randomly shifted -3~3 pixels lesion location as the center; (5) Extracted the three orthogonal scanning planes through the center of the lesion and stack them as pseudo-RGB images with size of 3*150*150; (6) Randomly cropped the stacks to 132*132; (7) Randomly cropped and resize the stacks to 112*112; (8) Randomly sharpen the stacks; (9) Randomly blurred the stacks; (10) Subtracted the mean and divide the variance. In addition, since the number of negative samples was much more than the number of positive samples in the dataset, we randomly up-sampled the positive ones at the beginning of each training epoch so that the ratio of positive to negative samples was kept at 1:1.

Supplementary Method 6. Neural network training process

Two separate models were trained to predict N1 and N2 metastasis so as to calculate separate scores. In N1 prediction, data were divided into N1 metastasis and non-N1 metastasis. Similarly, in N2 prediction, data were divided into N2 metastasis and non-N2 metastasis. In order to obtain the occult N1/N2 metastasis probability, we constructed a neural network model based on the ResNet-18 algorithm[8]. The model consisted of two parts: the feature extractor and the feature classifier. The input of the feature extractor was a tensor of size 3*112*112, and the main structure of this part was ResNet-18 which contains 8 residual blocks. The pretrained ResNet-18 based on ImageNet dataset [9] was utilized, which enabled better parameter initialization for medical image analysis tasks. The latter part classified the output map of the feature extractor and output two prediction probabilities, which represented the probability of metastasis and the probability of non-metastasis (sum to 1). The classifiers consisted of two fully-connected layers, the first layer had 512 output nodes, and the second one contained two nodes whose output values were calculated by the softmax function and converted into probabilities [7,10,11].

Additionally, for the deep learning nodal metastasis signature (DLNMS), we used two ResNet-18's feature extractors to extract the features from PET and CT, respectively. Then we combined PET and CT features as the following classifier's input. Models were trained with a mini-batch size of 32. We started training with a learning rate of 0.001 using CosineAnnealingWarmRestarts learning rate scheduler[12], and the restart epoch was set at 50. The entire network was trained by the adamw optimizer [13,14] for 100 epochs. We used the MixUp algorithm [15] as an additional data augmentation method. For the loss function, we used the binary focal loss function [16]. The code implementation was based on the pytorch [17]. The training of the algorithm was performed on a computer with a NVIDA 3090.

Over the training iteration, the loss in the training set decreased consistently and plateaued finally. In addition, the performance metrics in the validation set stabilized and shown only minor fluctuations. No overfitting or underfitting was observed in the training process. Above results indicated the DLNMS reached convergence. To evaluate the model computational requirements, parameters and floating point operations (FLOPs) were calculated, the number of parameters and FLOPs for the DLNMS were 24.47M and 974.88M Mac, respectively.

Supplementary Method 7. Selection for deep learning architecture, fusion strategy, and image augmentation

To select the optimal deep learning architecture, preliminary experiments were performed between ResNet-18, ResNet-50, ResNet-152, and DenseNet-121[8,18], revealing that deeper ResNets were easily overfitting than ResNet-18 and DenseNet-121 achieved a slightly lower performance than ResNet-18. Therefore, ResNet-18 was chosen as the basis of the DLNMS. For the fusion strategies of PET and CT images, the following three methods: input-level concatenation, feature-level combination, and output-level average were investigated, and

feature-level combination achieved the best performance and was therefore applied in the final DLNMS. For image augmentations, preliminary experiments were conducted to investigate the addition of a certain augmentation method would improve model performances, we primarily attempted several augmentation methods, revealing that the current augmentation flow consisted of random rotation, random shift, random crop, three orthogonal planes extraction, random sharpness, and random blur could increase the effective size of training data and alleviate the overfitting problem. In addition, transforms generated from the current augmentations could improve the generalization ability, which was beneficial for external validations.

Supplementary Method 8. Adjustments among multiple facilities

Three methods were utilized to standardize images. Firstly, for the original PET/CT images, the attenuation correction was applied to reduce statistical noises of original images and the corrected images were reconstructed for quantitative analyses. Secondly, to correct variability related to voxel size, each voxel in images was resampled to 0.6*0.6*0.6 mm3 in the spatial dimension. Finally, to reduce the batch effect error, pixel-wise ROI image data were subtracted by the mean intensity value and divided by the standard deviation of ROI image intensity.

Supplementary Method 9. Benchmarking

For the clinical model, multivariable logistic analyses were conducted to identified predictors for occult nodal metastasis (ONM) in the training set and clinical models were developed based predictors and their coefficients. For physicians, 3 senior radiologists with more than 10 years of experiences and 3 junior radiologists with less than 5 years of experiences were required to classify the ONM status based on imaging data at s setting blinded to pathological results. Patients suspected by physicians as ONM were scored as “1” and those considered as benign were scored as “0”. After all physicians completed evaluations for a given patient, the final scores for this patient of the senior and junior groups were calculated as the mean values of the 3 senior physicians and 3 junior physicians, respectively.

Supplementary Method 10. ONM scores of patients receiving limited nodal dissection (LND)

In clinical practice, LND and SND are both optional strategies in the treatments of early-stage NSCLC, but LND dissects fewer lymph nodes than SND. Therefore, to ensure that the algorithm could learn accurate nodal staging information, we primarily excluded patients receiving LND and only those receiving SND were included in the procedures for model construction and efficiency validation. However, to explore the value of DLNMS in guiding treatment decisions of early-stage NSCLC, we must compare the prognosis after different treatments in DLNMS defined risk groups, which indicates that it is necessary to obtain the ONM scores of patients receiving LND. In such instances, after the DLNMS was constructed and validated, PET/CT images of patients receiving LND were input into the DLNMS and their ONM scores were calculated. Subsequently, LND patients with predicted ONM scores by the DLNMS were reincluded in the prognostic analyses.

Supplementary Method 11. Gene set enrichment analysis

An adult NSCLC RNA-seq dataset was obtained from the Gene Expression Omnibus. Fragments Per Kilobase of transcript per million mapped reads were transformed to log2. Samples from 144 patients with NSCLC in the radiogenomics dataset were retrieved. According to the N1 cutoff values (score=0.362), these patients were divided into 129 patients with low N1 scores and 15 patients with high N1 scores. Similarly, based on the N2 cutoff values (score=0.356), these patients were divided into 126 patients with low N2 scores and 18 patients with high N2 scores. To identify differential expression genes between different groups, we applied the R package limma to fit linear models. After using a fold change to rank these genes as input, the gene set enrichment analysis was performed with the ReactomePA and clusterProfiler R packages. Gene sets were limited to sizes between 15 and 200 and the enrichment score was applied to quantify the association of the rank of genes with pathways.

Supplementary Method 12. Single sample gene set enrichment analysis

Samples from 144 patients with NSCLC in the radiogenomics dataset were retrieved. According to the N1 cutoff values (score=0.362), these patients were divided into 129 patients with low N1 scores and 15 patients with high N1 scores. Similarly, based on the N2 cutoff values (score=0.356), these patients were divided into 126 patients with low N2 scores and 18 patients with high N2 scores. The single sample gene set enrichment analysis was conducted with the GSVA R package to quantify the relative infiltration of 28 immune cell types in the tumor microenvironment. Feature gene panels for each immune cell type were obtained from a previous publication (PMID: 28052254). The relative abundance of each immune cell type was represented by an enrichment score in the single sample gene set enrichment analysis. The enrichment score was normalized to unity distribution, for which zero is the minimal and one is the maximal score for each immune cell type. The bio-similarity of the immune cell filtration was estimated by multidimensional scaling and a Gaussian fitting model. Finally, infiltrations of immune cells in the tumor microenvironment were compared between two groups.

Supplementary Table 1. Logistic analyses of occult N1 and N2 metastasis before incorporation of the DLNMS for patients in the validation set, external cohort and prospective cohort

Variables	Occult N1				Occult N2			
	Univariable		Multivariable		Univariable		Multivariable	
	OR (95% CI)	p value	OR (95% CI)	adjusted p value	OR (95% CI)	p value	OR (95% CI)	adjusted p value
Age	0.985 (0.970-1.001)	0.066	0.969 (0.952-0.986)	<0.001	1.010 (0.991-1.029)	0.318		
Sex (Male)	1.885 (1.396-2.544)	<0.001	2.110 (1.489-2.989)	<0.001	0.940 (0.671-1.317)	0.720		
Smoking history (Ever)	1.123 (0.733-1.305)	0.799			1.117 (0.821-1.203)	0.841		
Radiological type (Solid)	4.810 (3.240-7.141)	<0.001	6.886 (5.274-9.101)	<0.001	3.161 (2.092-4.776)	<0.001	8.697 (4.559-16.591)	<0.001
Location (Left)	1.149 (0.892-1.599)	0.234			1.126 (0.803-1.579)	0.490		
Location (Central)	2.962 (2.157-4.068)	<0.001	0.950 (0.647-1.395)	0.952	1.516 (1.019-2.255)	0.040	1.419 (1.172-1.718)	0.070
Tumor size	1.258 (1.150-1.376)	<0.001	1.084 (0.965-1.217)	0.261	1.159 (1.047-1.284)	0.004	1.097 (0.949-1.269)	0.281
SUVmax	1.114 (1.086-1.142)	<0.001	0.960 (0.700-1.317)	0.800	1.085 (1.055-1.115)	<0.001	0.800 (0.529-1.211)	0.291
MTV	0.997 (0.987-1.007)	0.569			0.989 (0.959-1.022)	0.552		
TLG	1.000 (1.000-1.001)	0.300			1.000 (0.999-1.001)	0.805		

DLNMS, deep learning nodal metastasis signature; SUV, standard uptake value; MTV, metabolic tumor volume; TLG, total lesion glycolysis; HR, hazard ratio: CI, confidence interval; p values of multivariable analyses were corrected by the Benjamini and Hochberg method.

Supplementary Table 2. Logistic analyses of occult N1 and N2 metastasis after incorporation of the DLNMS for patients in the training set

Variables	Occult N1				Occult N2			
	Univariable		Multivariable		Univariable		Multivariable	
	OR (95% CI)	p value	OR (95% CI)	adjusted p value	OR (95% CI)	p value	OR (95% CI)	adjusted p value
Age	0.979 (0.963-0.994)	0.008	0.972 (0.946-1.000)	0.147	0.992 (0.973-1.012)	0.445		
Sex (Male)	1.929 (1.404-2.652)	<0.001	0.939 (0.525-1.677)	0.935	1.463 (1.008-2.125)	0.046	0.936 (0.589-1.488)	0.936
Smoking history (Ever)	1.152 (0.824-1.470)	0.855			1.301 (0.878-1.353)	0.765		
Radiological type (Solid)	4.005 (2.718-5.903)	<0.001	0.597 (0.271-1.317)	0.362	4.231 (2.614-6.848)	<0.001	1.089 (0.549-2.161)	0.807
Location (Left)	1.429 (1.048-1.949)	0.024	1.017 (0.595-1.737)	0.952	1.249 (0.862-1.810)	0.241		
Location (Central)	2.998 (2.146-4.188)	<0.001	0.568 (0.254-1.063)	0.259	1.936 (1.282-2.924)	0.002	0.726 (0.440-1.197)	0.630
Tumor size	1.385 (1.239-1.548)	<0.001	1.370 (1.086-1.727)	0.036	1.269 (1.124-1.433)	<0.001	1.040 (0.862-1.254)	0.999
SUVmax	1.127 (1.095-1.160)	<0.001	0.912 (0.598-1.391)	0.861	1.094 (1.060-1.129)	<0.001	0.842 (0.474-1.368)	0.848
MTV	1.001 (0.993-1.010)	0.746			0.999 (0.986-1.011)	0.845		
TLG	1.001 (1.000-1.001)	0.065	1.001 (0.999-1.002)	0.429	1.000 (1.000-1.001)	0.242		
DLNMS	1307.850 (593.604-2881.506)	<0.001	2382.108 (885.311-6409.545)	<0.001	128.017 (64.132-255.540)	<0.001	206.552 (92.735-460.062)	<0.001

DLNMS, deep learning nodal metastasis signature; SUV, standard uptake value; MTV, metabolic tumor volume; TLG, total lesion glycolysis; HR, hazard ratio: CI, confidence interval; p values of multivariable analyses were corrected by the Benjamini and Hochberg method.

Supplementary Table 3. Logistic analyses of occult N1 and N2 metastasis after incorporation of the DLNMS for patients in the validation set, external cohort and prospective cohort

Variables	Occult N1				Occult N2			
	Univariable		Multivariable		Univariable		Multivariable	
	OR (95% CI)	p value	OR (95% CI)	adjusted p value	OR (95% CI)	p value	OR (95% CI)	adjusted p value
Age	0.985 (0.970-1.001)	0.066	0.975 (0.952-0.999)	0.157	1.010 (0.991-1.029)	0.318		
Sex (Male)	1.885 (1.396-2.544)	<0.001	1.586 (0.968-2.601)	0.156	0.940 (0.671-1.317)	0.720		
Smoking history (Ever)	1.123 (0.733-1.305)	0.799			1.117 (0.821-1.203)	0.841		
Radiological type (Solid)	4.810 (3.240-7.141)	<0.001	1.066 (0.535-2.152)	0.842	3.161 (2.092-4.776)	<0.001	1.949 (0.655-2.774)	0.648
Location (Left)	1.149 (0.892-1.599)	0.234			1.126 (0.803-1.579)	0.490		
Location (Central)	2.962 (2.157-4.068)	<0.001	0.646 (0.208-1.452)	0.357	1.516 (1.019-2.255)	0.040	0.572 (0.214-1.147)	0.288
Tumor size	1.258 (1.150-1.376)	<0.001	1.155 (0.980-1.360)	0.149	1.159 (1.047-1.284)	0.004	1.004 (0.832-1.210)	0.971
SUVmax	1.114 (1.086-1.142)	<0.001	0.971 (0.642-1.468)	0.888	1.085 (1.055-1.115)	<0.001	0.703 (0.428-1.156)	0.275
MTV	0.997 (0.987-1.007)	0.569			0.989 (0.959-1.022)	0.552		
TLG	1.000 (1.000-1.001)	0.300			1.000 (0.999-1.001)	0.805		
DLNMS	508.761 (274.343-943.480)	<0.001	435.448 (216.951-873.998)	<0.001	69.339 (39.648-121.266)	<0.001	66.550 (36.697-120.689)	<0.001

DLNMS, deep learning nodal metastasis signature; SUV, standard uptake value; MTV, metabolic tumor volume; TLG, total lesion glycolysis; HR, hazard ratio: CI, confidence interval; p values of multivariable analyses were corrected by the Benjamini and Hochberg method.

Supplementary Table 4. Integrated discrimination improvement and net reclassification index of DLNMS for occult N1 and N1 prediction

Tasks	Comparison	Cohort	IDI	adjusted p value	NRI (categorical)	adjusted p value	NRI (continuous)	adjusted p value
Occult N1	DLNMS versus PET	Training set	0.231 [0.193-0.268]	<0.001	0.236 [0.160-0.312]	<0.001	1.237 [1.099-1.379]	<0.001
Occult N1	DLNMS versus PET	Validation set	0.279 [0.201-0.357]	<0.001	0.368 [0.194-0.542]	<0.001	1.377 [1.136-1.619]	<0.001
Occult N1	DLNMS versus PET	External cohort	0.238 [0.138-0.338]	<0.001	0.223 [0.020-0.426]	0.031	0.991 [0.679-1.302]	<0.001
Occult N1	DLNMS versus PET	Prospective cohort	0.260 [0.214-0.305]	<0.001	0.308 [0.211-0.405]	<0.001	1.206 [1.033-1.379]	<0.001
Occult N1	DLNMS versus CT	Training set	0.181 [0.146-0.216]	<0.001	0.166 [0.093-0.240]	<0.001	1.042 [0.894-1.190]	<0.001
Occult N1	DLNMS versus CT	Validation set	0.211 [0.143-0.279]	<0.001	0.305 [0.153-0.458]	<0.001	1.029 [0.748-1.311]	<0.001
Occult N1	DLNMS versus CT	External cohort	0.175 [0.089-0.262]	<0.001	0.305 [0.082-0.528]	0.007	1.003 [0.693-1.314]	<0.001
Occult N1	DLNMS versus CT	Prospective cohort	0.216 [0.175-0.256]	<0.001	0.286 [0.195-0.377]	<0.001	1.118 [0.939-1.297]	<0.001
Occult N2	DLNMS versus PET	Training set	0.450 [0.393-0.508]	<0.001	0.666 [0.546-0.786]	<0.001	1.385 [1.247-1.524]	<0.001
Occult N2	DLNMS versus PET	Validation set	0.471 [0.353-0.590]	<0.001	0.729 [0.481-0.977]	<0.001	1.33 [1.029-1.635]	<0.001
Occult N2	DLNMS versus PET	External cohort	0.349 [0.209-0.489]	<0.001	0.569 [0.312-0.826]	<0.001	1.021 [0.697-1.345]	<0.001
Occult N2	DLNMS versus PET	Prospective cohort	0.462 [0.391-0.532]	<0.001	0.684 [0.542-0.827]	<0.001	1.374 [1.208-1.540]	<0.001
Occult N2	DLNMS versus CT	Training set	0.220 [0.162-0.278]	<0.001	0.412 [0.337-0.486]	<0.001	0.691 [0.514-0.868]	<0.001
Occult N2	DLNMS versus CT	Validation set	0.291 [0.156-0.426]	<0.001	0.502 [0.315-0.690]	<0.001	0.849 [0.482-1.218]	<0.001
Occult N2	DLNMS versus CT	External cohort	0.206 [0.075-0.338]	0.002	0.246 [0.056-0.437]	0.011	0.621 [0.282-0.960]	<0.001
Occult N2	DLNMS versus CT	Prospective cohort	0.345 [0.257-0.433]	<0.001	0.477 [0.342-0.612]	<0.001	1.295 [1.124-1.466]	<0.001

DLNMS, deep learning nodal metastasis signature; IDI, Integrated discrimination improvement; NRI, net reclassification index; CI, confidence interval; p values were corrected by the Benjamini and Hochberg method.

Supplementary Table 5. Baseline characteristics of patients receiving nodal biopsy

Characteristics	n=366
Age (years)	
>65, n (%)	130 (35.52)
≤65, n (%)	236 (64.48)
Mean ± SD	61.28 ± 9.27
Sex, n (%)	
Male	256 (69.95)
Female	110 (30.05)
Smoking, n (%)	
Ever	114 (31.14)
Never	252 (68.86)
Radiologic type, n (%)	
Pure solid	334 (91.26)
Subsolid	32 (8.74)
PET parameters	
SUVmax, mean ± SD	10.76 ± 5.55
MTV, mean ± SD	19.65 ± 32.29
TLG, mean ± SD	159.61 ± 431.80
Location, n (%)	
Left	151 (41.26)
Right	215 (58.74)
Central	271 (74.04)
Peripheral	95 (25.96)
Radiological size (cm), mean ± SD	4.10 ± 1.98
N2 involvement, n (%)	
Yes	74 (20.22)
No	292 (79.78)
Pathological type, n (%)	
Adenocarcinoma	208 (56.83)
Squamous cell carcinoma	122 (33.33)
Others	36 (9.84)
Initial treatment modality, n (%)	
Upfront surgery	232 (63.39)
Neoadjuvant therapy followed by surgery	43 (11.75)
Chemotherapy/Targeted therapy/Immunotherapy	52 (14.21)
Radiotherapy	12 (3.28)
Chemoradiotherapy	27 (7.38)

PET, positron emission tomography; SUV, standard uptake value; MTV, metabolic tumor volume; TLG, total lesion glycolysis; SD, standard deviation.

Supplementary Table 6. Baseline characteristics of clinical stage I patients receiving limited lymph node dissection

Characteristics	n=654
Age (years)	
>65, n (%)	193 (29.51)
≤65, n (%)	461 (70.49)
Mean ± SD	59.40 ± 10.20
Sex, n (%)	
Male	287 (43.88)
Female	367 (56.12)
Smoking, n (%)	
Ever	71 (10.86)
Never	583 (89.14)
Radiologic lesion type, n (%)	
Pure solid	317 (48.47)
Subsolid	337 (51.53)
PET parameters	
SUVmax, mean ± SD	4.05 ± 4.12
MTV, mean ± SD	7.98 ± 7.36
TLG, mean ± SD	14.53 ± 19.97
Surgery procedure, n (%)	
Sublobectomy	575 (87.92)
Lobectomy	79 (12.08)
Location, n (%)	
Left	277 (42.35)
Right	377 (57.65)
Central	81 (12.39)
Peripheral	573 (87.61)
Tumor size (cm), mean ± SD	2.06 ± 0.73
N1 involvement, n (%)	
Yes	41 (6.27)
No	613 (93.73)
N2 involvement, n (%)	
Yes	18 (2.75)
No	636 (97.25)
Pathological type, n (%)	
Adenocarcinoma	586 (89.60)
Squamous cell carcinoma	44 (6.73)
Others	24 (3.67)

SUV, standard uptake value; MTV, metabolic tumor volume; TLG, total lesion glycolysis; SD, standard deviation.

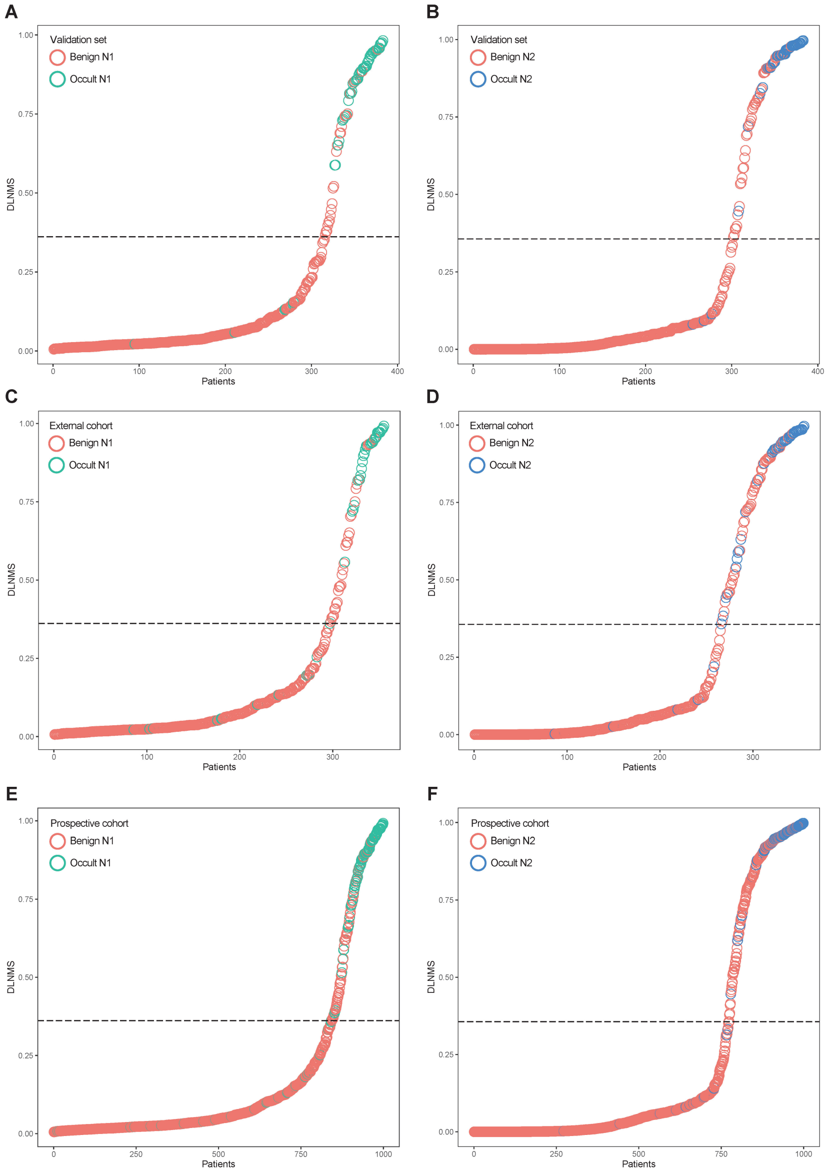

Supplementary Fig. 1. Scatter graphs illustrating the DLNMS score distributions in the (A & B) validation set, (C & D) external cohort, and (E & F) prospective cohort.
Source data are provided as a Source Data file. DLNMS, deep learning nodal metastasis signature.

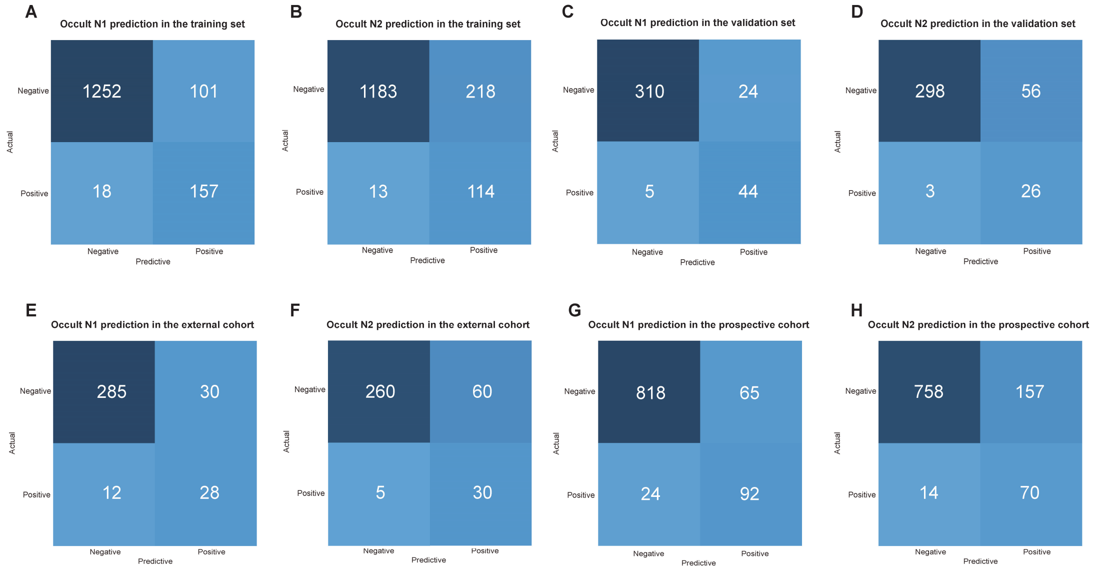

Supplementary Fig. 2. Confusion matrixes of the DLNMS to identify occult nodal metastasis in the (A & B) training set, (C & D) validation set, (E & F) external cohort and (G & H) prospective cohort.

Source data are provided as a Source Data file. DLNMS, deep learning nodal metastasis signature.

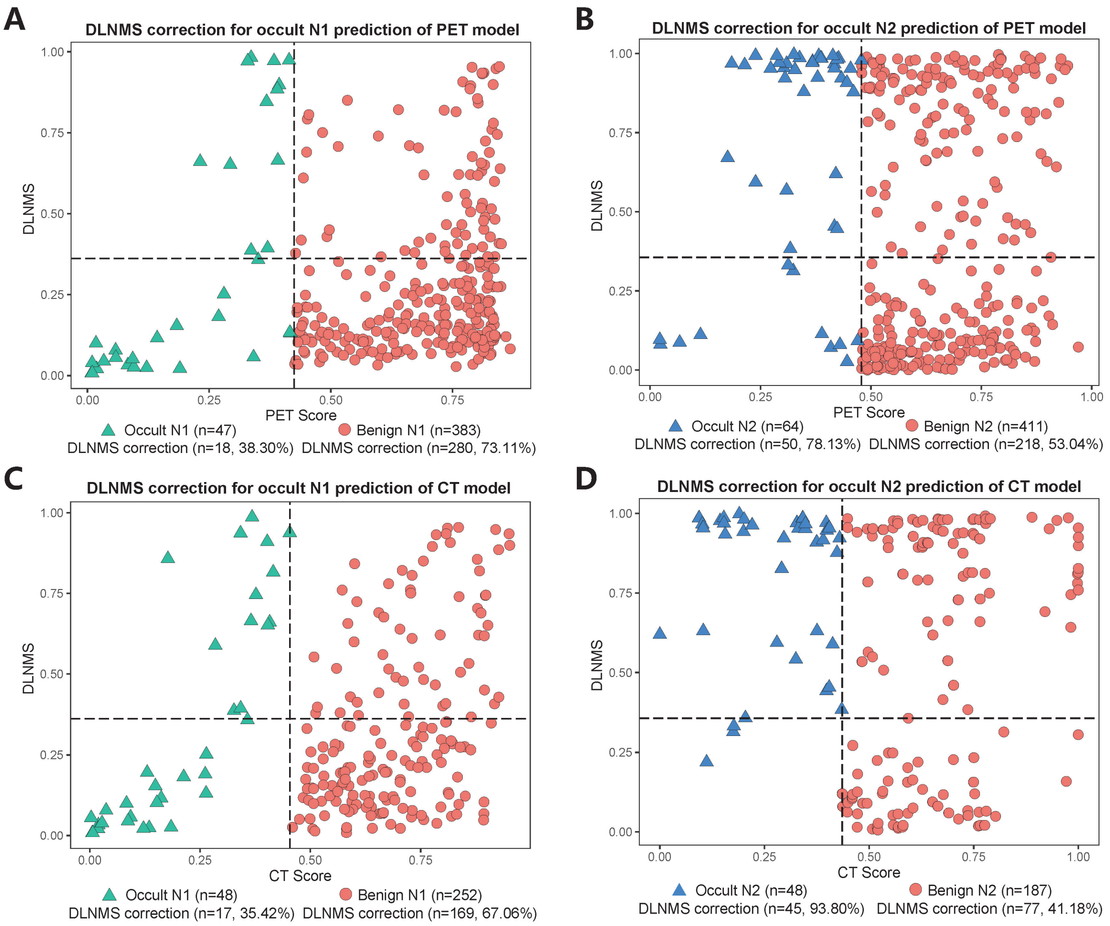

Supplementary Fig. 3. Scatter graphs illustrating the DLNMS correct cases falsely predicted by the (A & B) PET and (C & D) CT models in the validation set, external cohort and prospective cohort.

Source data are provided as a Source Data file. DLNMS, deep learning nodal metastasis signature; PET, positron emission tomography; CT, computed tomography.

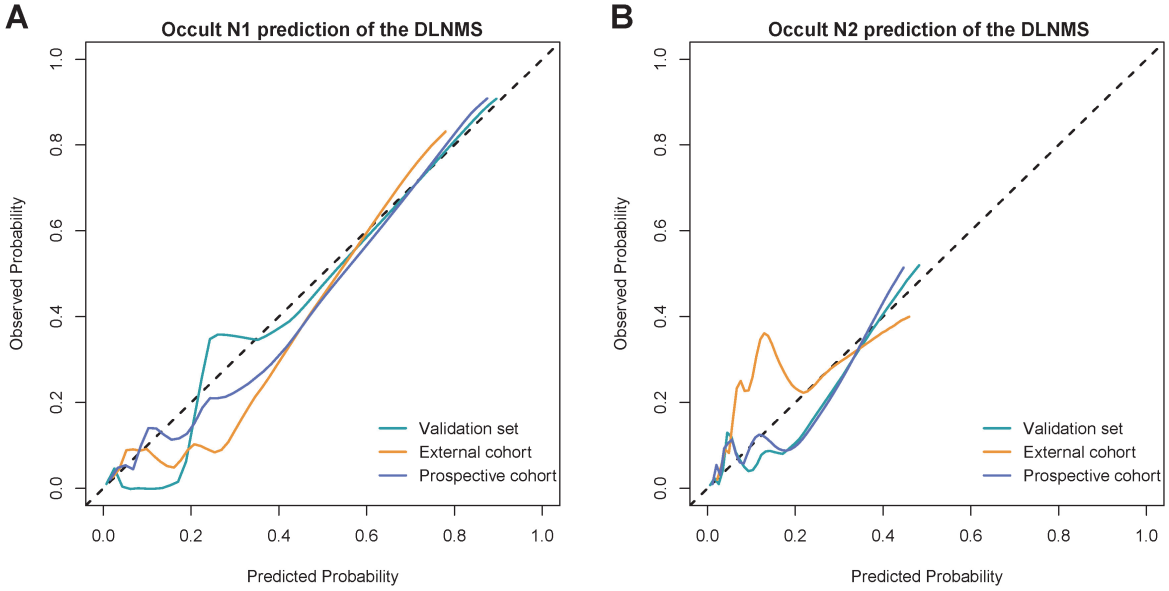

Supplementary Fig. 4. Calibration curves of the DLNMS to identify (A & B) occult N1 and (C & D) N2 metastasis.

Source data are provided as a Source Data file. DLNMS, deep learning nodal metastasis signature.

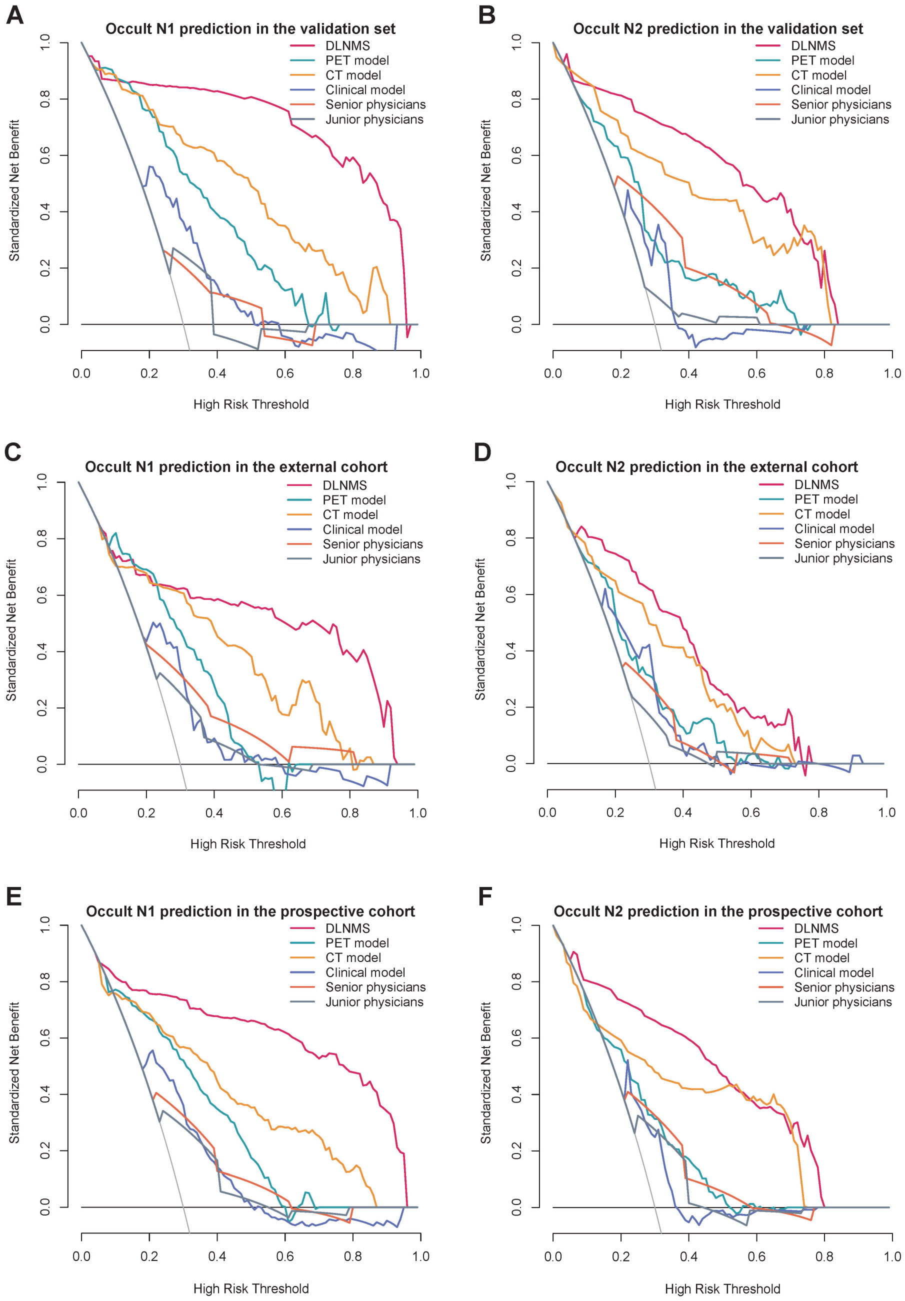

Supplementary Fig. 5. Decision curves of the DLNMS to identify occult nodal metastasis in the (A & B) validation set, (C & D) external cohort and (E & F) prospective cohort. Source data are provided as a Source Data file. DLNMS, deep learning nodal metastasis signature; PET, positron emission tomography; CT, computed tomography.

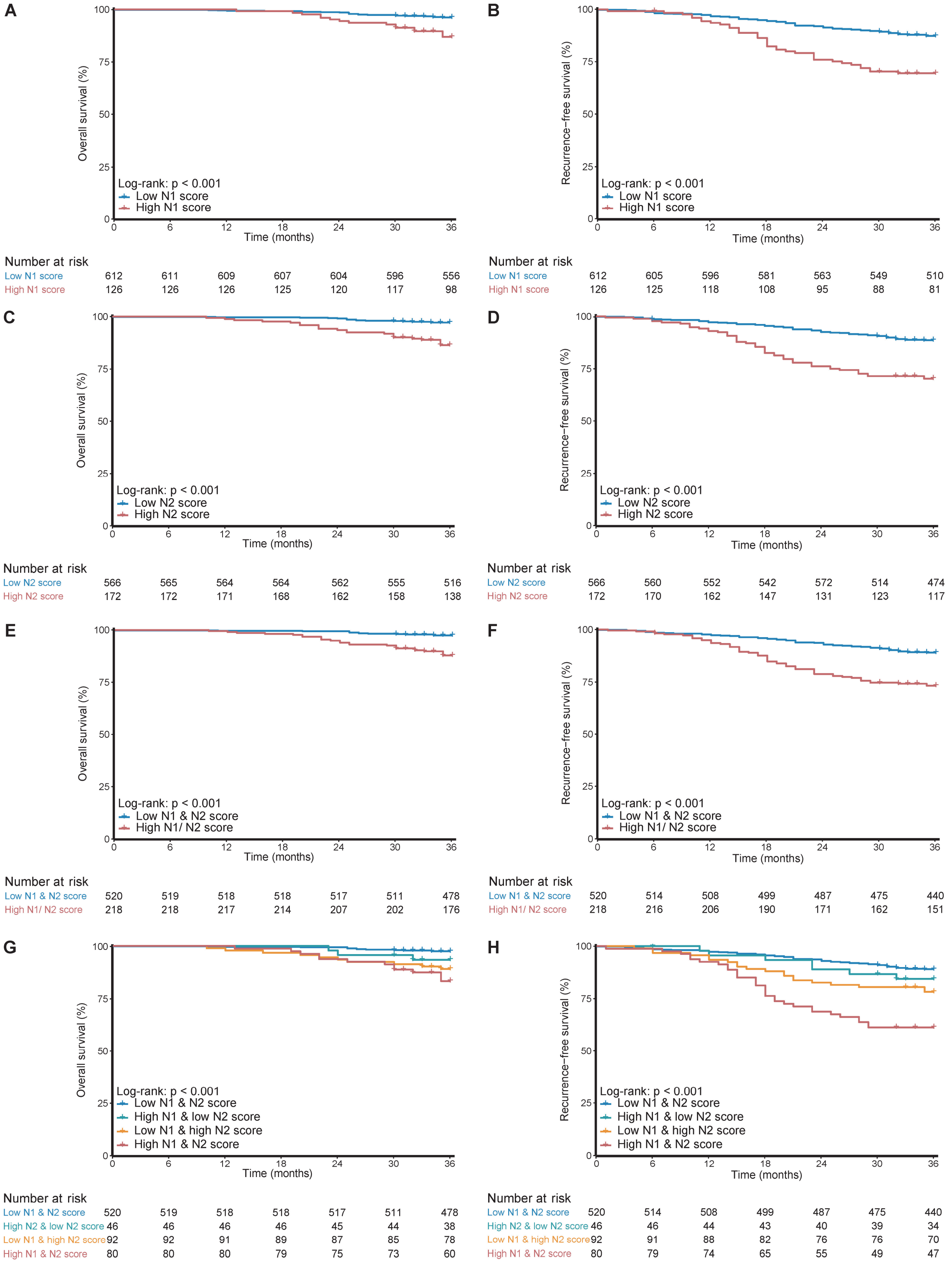

Supplementary Fig. 6. Prognosis of patients with non-small cell lung cancer grouped by different risk scores in the validation set and external cohort.

n=738 biologically independent samples were examined. Survival data were compared by the log-rank test. Source data are provided as a Source Data file.

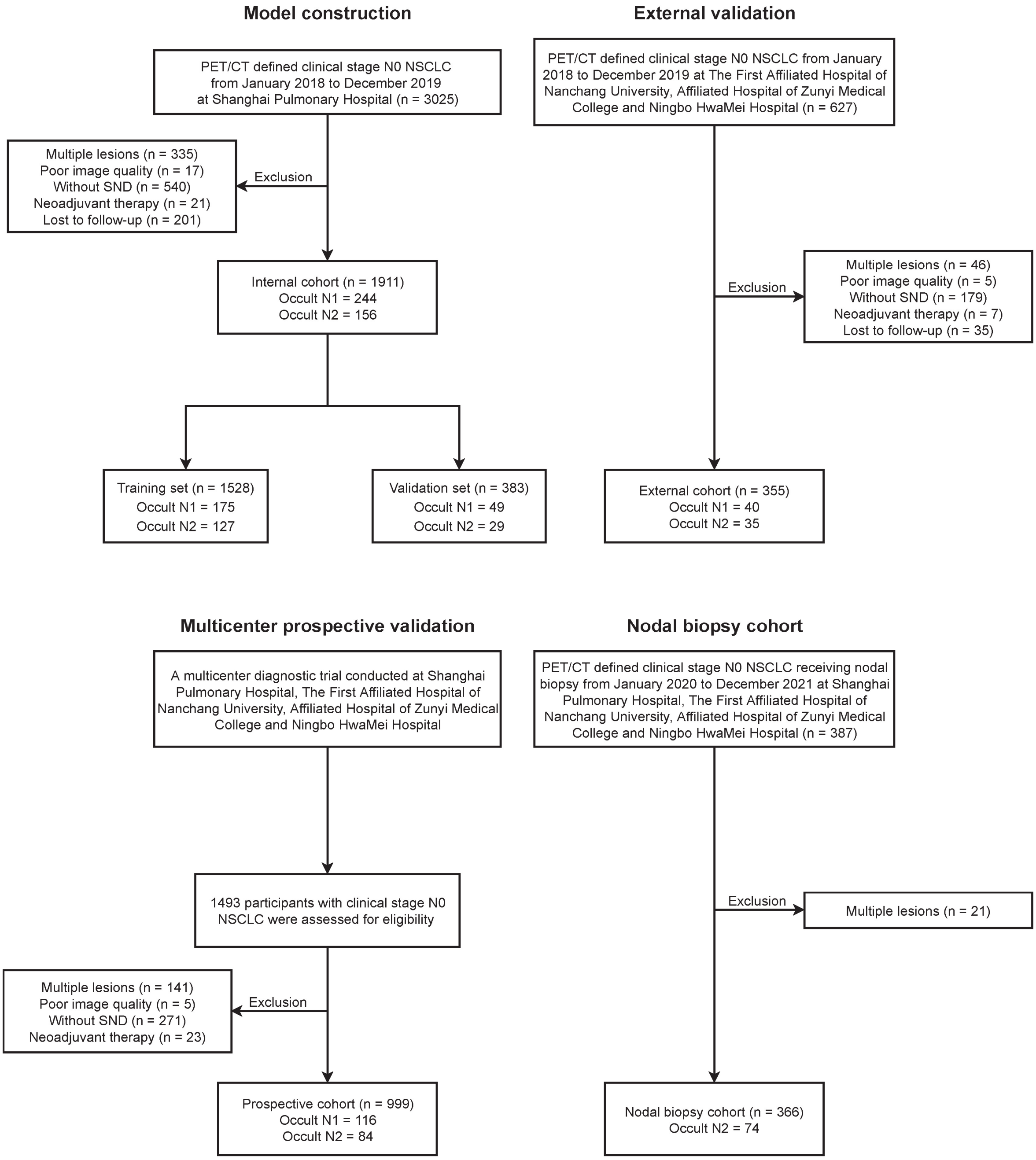

Supplementary Fig. 7. Flow chart illustrating patient selection.
PET/CT, positron emission tomography-computed tomography; NSCLC, non-small cell lung cancer; SND, systematic nodal dissection.

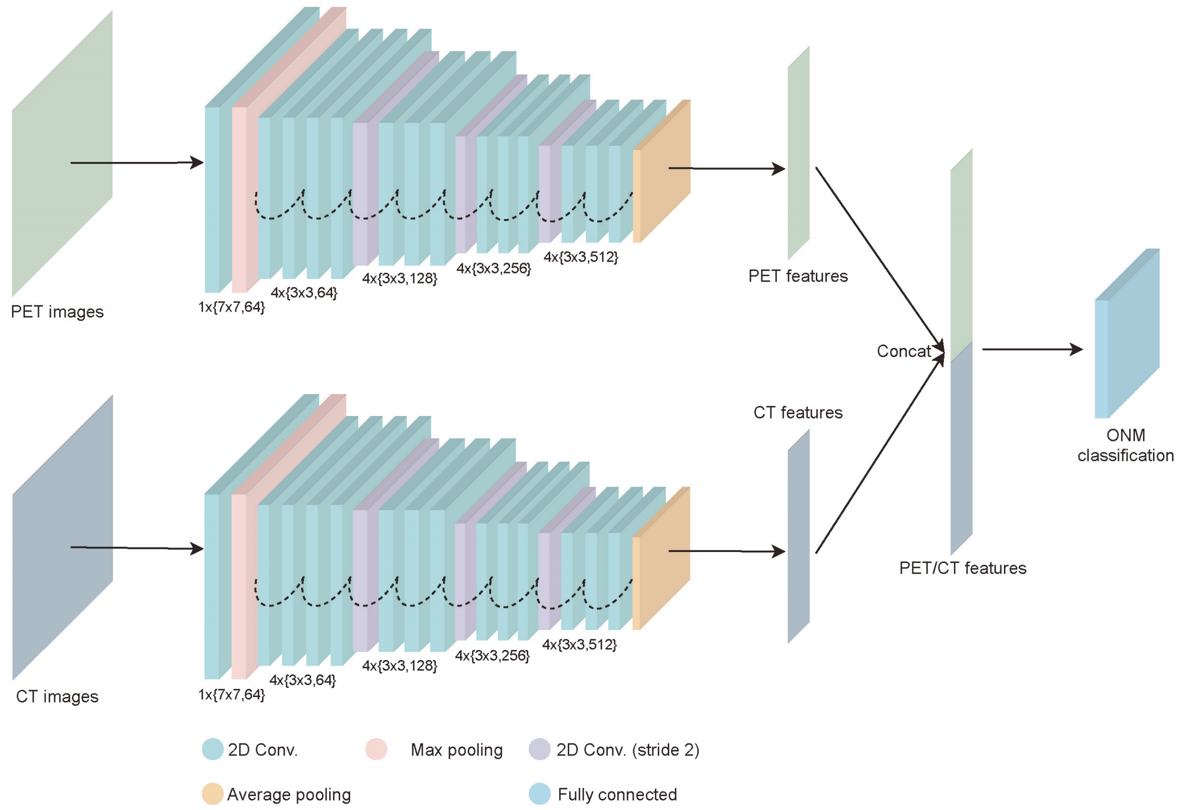

Supplementary Fig. 8. Diagram illustrating the structure of DLNMS.
DLNMS, deep learning nodal metastasis signature; PET, positron emission tomography; CT, computed tomography; ONM, occult nodal metastasis.

References

1 Hariharan, B., Arbeláez, P., Girshick, R. & Malik, J. Hypercolumns for object segmentation and fine-grained localization. *Proceedings of the IEEE conference on computer vision and pattern recognition*, 447-456 (2015).

2 Lee, K., Zung, J., Li, P., Jain, V. & Seung, H. S. Superhuman accuracy on the SNEMI3D connectomics challenge. *arXiv preprint arXiv:1706.00120* (2017).

3 Quan, T., Hildebrand, D. & Jeong, W. Fusionnet: A deep fully residual convolutional neural network for image segmentation in connectomics. arXiv 2016. *arXiv preprint arXiv:1612.05360* (2016).

4 Beier, T. *et al.* Multicut brings automated neurite segmentation closer to human performance. *Nature methods* **14**, 101-102 (2017).

5 Toubal, I. E., Duan, Y. & Yang, D. Deep learning semantic segmentation for high-resolution medical volumes. *2020 IEEE Applied Imagery Pattern Recognition Workshop (AIPR)*, 1-9 (2020).

6 Zeng, T., Wu, B. & Ji, S. DeepEM3D: approaching human-level performance on 3D anisotropic EM image segmentation. *Bioinformatics (Oxford, England)* **33**, 2555-2562 (2017).

7 Simonyan, K. & Zisserman, A. Very deep convolutional networks for large-scale image recognition. *arXiv preprint arXiv:1409.1556* (2014).

8 He, K., Zhang, X., Ren, S. & Sun, J. Deep residual learning for image recognition. *Proceedings of the IEEE conference on computer vision and pattern recognition*, 770-778 (2016).

9 Russakovsky, O. *et al.* Imagenet large scale visual recognition challenge. *International journal of computer vision* **115**, 211-252 (2015).

10 Goodfellow, I., Bengio, Y. & Courville, A. 6.2. 2.3 softmax units for multinoulli output distributions. *Deep learning* **180** (2016).

11 Brownlee, J. *Probability for machine learning: Discover how to harness uncertainty with Python*. (Machine Learning Mastery, 2019).

12 Loshchilov, I. & Hutter, F. Sgdr: Stochastic gradient descent with warm restarts. *arXiv preprint arXiv:1608.03983* (2016).

13 Loshchilov, I. & Hutter, F. Decoupled weight decay regularization. *arXiv preprint arXiv:1711.05101* (2017).

14 Loshchilov, I. & Hutter, F. Fixing weight decay regularization in adam. *arXiv preprint arXiv:1711.05101* (2018).

15 Zhang, H., Cisse, M., Dauphin, Y. N. & Lopez-Paz, D. mixup: Beyond empirical risk minimization. *arXiv preprint arXiv:1710.09412* (2017).

16 Lin, T.-Y., Goyal, P., Girshick, R., He, K. & Dollár, P. Focal loss for dense object detection. *Proceedings of the IEEE international conference on computer vision*, 2980-2988 (2017).

17 Paszke, A. *et al.* Pytorch: An imperative style, high-performance deep learning library. *Advances in neural information processing systems* **32** (2019).

18 Huang, G., Liu, Z., Van Der Maaten, L. & Weinberger, K. Q. Densely connected convolutional networks. *Proceedings of the IEEE conference on computer vision and pattern recognition*, 4700-4708 (2017).
