# Supplementary material for: PET/CT based cross-modal deep learning signature to predict occult nodal metastasis in lung cancer
Source: Nat Commun. 2023 Nov 18;14:7513. doi: 10.1038/s41467-023-42811-4 (PMC10657428; doi:10.1038/s41467-023-42811-4)
Supplement: Supplementary file 3 — Reporting Summary [file 41467_2023_42811_MOESM3_ESM.pdf]

nature portfolio

Corresponding author(s): Chang Chen

Last updated by author(s): 2023-9-13

Reporting Summary

Nature Portfolio wishes to improve the reproducibility of the work that we publish. This form provides structure for consistency and transparency in reporting. For further information on Nature Portfolio policies, see our Editorial Policies and the Editorial Policy Checklist.

Statistics

For all statistical analyses, confirm that the following items are present in the figure legend, table legend, main text, or Methods section.

n/a	Confirmed	
☐	☒	The exact sample size (*n*) for each experimental group/condition, given as a discrete number and unit of measurement
☐	☒	A statement on whether measurements were taken from distinct samples or whether the same sample was measured repeatedly
☐	☒	The statistical test(s) used AND whether they are one- or two-sided<br>*Only common tests should be described solely by name; describe more complex techniques in the Methods section.*
☐	☒	A description of all covariates tested
☐	☒	A description of any assumptions or corrections, such as tests of normality and adjustment for multiple comparisons
☐	☒	A full description of the statistical parameters including central tendency (e.g. means) or other basic estimates (e.g. regression coefficient) AND variation (e.g. standard deviation) or associated estimates of uncertainty (e.g. confidence intervals)
☐	☒	For null hypothesis testing, the test statistic (e.g. *F*, *t*, *r*) with confidence intervals, effect sizes, degrees of freedom and *P* value noted<br>*Give P values as exact values whenever suitable.*
☒	☐	For Bayesian analysis, information on the choice of priors and Markov chain Monte Carlo settings
☐	☒	For hierarchical and complex designs, identification of the appropriate level for tests and full reporting of outcomes
☐	☒	Estimates of effect sizes (e.g. Cohen's *d*, Pearson's *r*), indicating how they were calculated

Our web collection on statistics for biologists contains articles on many of the points above.

Software and code

Policy information about availability of computer code

Data collection	All PET/CT images were registered and segmented with 3D slicer software (version 4.8.0, Brigham and Women's Hospital). Data processing was conducted using Python (version 3.10, https://www.python.org/). All computer codes for preprocessing and training are summarized at https://github.com/zhongthoracic/DLNMS.
Data analysis	The process and method of data analysis are discussed in detail in the method section of the article. Data analysis was conducted using SPSS (version 25.0, IBM SPSS Statistics), R program (version 4.1.3, http://www.Rproject.org) and Python (version 3.10, https://www.python.org/). All computer codes for preprocessing and training are summarized at https://github.com/zhongthoracic/DLNMS.

For manuscripts utilizing custom algorithms or software that are central to the research but not yet described in published literature, software must be made available to editors and reviewers. We strongly encourage code deposition in a community repository (e.g. GitHub). See the Nature Portfolio guidelines for submitting code & software for further information.

Data

Policy information about availability of data

All manuscripts must include a data availability statement. This statement should provide the following information, where applicable:

- Accession codes, unique identifiers, or web links for publicly available datasets
- A description of any restrictions on data availability
- For clinical datasets or third party data, please ensure that the statement adheres to our policy

Source data are provided with this paper. However, considering imaging data contain private information of patients, the PET/CT imaging data in the internal

cohort, external cohort, prospective cohort and biopsy are not publicly available for patient privacy purposes but are available from the corresponding authors upon reasonable request.

Human research participants

Policy information about studies involving human research participants and Sex and Gender in Research.

Reporting on sex and gender	We use the terms sex based on the biological attribute of participants. No finding in this study apply to only one sex or gender. Sex and gender were not considered in the study design. The overall number of this study was 3265 (male: 1587; female: 1678).
Population characteristics	The mean age of the entire cohort was 60·00 years and 48·61% (n=1587) of the population were male. There were 2776 (85·02%) adenocarcinomas and 340 (10·41%) squamous cell carcinomas. The maximum standard uptake value (SUVmax), metabolic tumor volume (MTV), total lesion glycolysis (TLG) of the primary tumors were 5·43, 10·13 and 37·74, respectively. With respect to N status, 11·64% (n=380) and 8·42% (n=275) of patients were diagnosed as occult N1 and N2 diseases. In addition, compared to the internal cohort, patients in the external cohort were associated with significantly and older age (61·78 years versus 59·42 years, p<0·001) and patients in the prospective cohort yielded an older age (60·46 years versus 59·42 years, p=0·005), higher SUVmax of primary tumor (5·67 versus 5·25, p=0·022) and larger tumor size (2·64 cm versus 2·53 cm, p=0·030).
Recruitment	In the internal cohort, consecutive patients receiving curative resection for clinical stage N0 non-small cell lung cancer (NSCLC) from January 2018 to December 2019 at Shanghai Pulmonary Hospital were retrospectively reviewed. Criteria for inclusion were as follows: (a) patients receiving curative surgery for primary NSCLC; (b) the maximum short-axis diameter of N1 and N2 lymph nodes less than 1 cm on CT scan; (c) the maximum standardized uptake value (SUVmax) of N1 and N2 lymph nodes less than 2·5. Criteria for exclusion included (a) multiple lung lesions; (b) poor quality of positron emission tomography/computed tomography (PET/CT) images; (c) patient not receiving systematic nodal dissection (SND); (d) patient receiving neoadjuvant therapy; and (e) lost to follow-up.<br><br>In the external cohort, we retrospectively included patients receiving curative resection for clinical stage N0 NSCLC from January 2018 to December 2019 at The First Affiliated Hospital of Nanchang University, Affiliated Hospital of Zunyi Medical College and Ningbo HwaMei Hospital. Criteria for inclusion were as follows: (a) patients receiving curative surgery for primary NSCLC; (b) the maximum short-axis diameter of N1 and N2 nodes less than 1 cm on CT scan; (c) the SUVmax of N1 and N2 lymph nodes less than 2·5. Criteria for exclusion included (a) multiple lung lesions; (b) poor quality of PET/CT images; (c) patient not receiving SND; (d) patient receiving neoadjuvant therapy; and (e) lost to follow-up.<br><br>In the prospective cohort, participants receiving curative resection for clinical stage N0 NSCLC from January 2022 to December 2022 at Shanghai Pulmonary Hospital, The First Affiliated Hospital of Nanchang University, Affiliated Hospital of Zunyi Medical College and Ningbo HwaMei Hospital were enrolled. Criteria for enrollment were as follows: (a) participants scheduled for surgery for radiological finding of pulmonary lesions from the preoperative thin-section CT scans; (b) the maximum short-axis diameter of N1 and N2 lymph nodes less than 1 cm on CT scan; (c) the SUVmax of N1 and N2 lymph nodes less than 2·5; (d) pathological confirmation of primary NSCLC; (e) age ranging from 20-75 years; and (f) obtained written informed consent. Criteria for exclusion included (a) multiple lung lesions; (b) poor quality of PET/CT images; (c) participants with incomplete clinical information; (d) participants not receiving SND and (e) participants receiving neoadjuvant therapy.<br><br>In the biopsy cohort, patients receiving nodal biopsy for clinical stage N0 NSCLC from January 2020 to December 2021 at Shanghai Pulmonary Hospital, The First Affiliated Hospital of Nanchang University, Affiliated Hospital of Zunyi Medical College and Ningbo HwaMei Hospital were retrospective reviewed. Criteria for inclusion were as follows: (a) patients receiving endobronchial ultrasound transbronchial needle aspirations for primary NSCLC; (b) the maximum short-axis diameter of N1 and N2 lymph nodes less than 1 cm on CT scan; and (c) the SUVmax of N1 and N2 lymph nodes less than 2·5. Patients with multiple lung lesions were excluded.
Ethics oversight	This study was implemented under the approval of the Institutional Review Board of Shanghai Pulmonary Hospital, The First Affiliated Hospital of Nanchang University, Affiliated Hospital of Zunyi Medical College and Ningbo HwaMei Hospital.

Note that full information on the approval of the study protocol must also be provided in the manuscript.

Field-specific reporting

Please select the one below that is the best fit for your research. If you are not sure, read the appropriate sections before making your selection.

[x] Life sciences [ ] Behavioural & social sciences [ ] Ecological, evolutionary & environmental sciences

For a reference copy of the document with all sections, see nature.com/documents/nr-reporting-summary-flat.pdf

Life sciences study design

All studies must disclose on these points even when the disclosure is negative.

Sample size	The internal cohort, the external cohort and the biopsy cohort were retrospectively collected. The prospective cohort was based on a prospective observational trial. Therefore, no sample size calculation was performed. This study is the largest study to predict ONM of NSCLC. Therefore, we think these sample sizes are sufficient.
Data exclusions	In the internal cohort, criteria for inclusion were as follows: (a) patients receiving curative surgery for primary NSCLC; (b) the maximum short-axis diameter of N1 and N2 lymph nodes less than 1 cm on CT scan; (c) the maximum standardized uptake value (SUVmax) of N1 and N2 lymph nodes less than 2·5. Criteria for exclusion included (a) multiple lung lesions; (b) poor quality of positron emission tomography/ computed tomography (PET/CT) images; (c) patient not receiving systematic nodal dissection (SND); (d) patient receiving neoadjuvant therapy; and (e) lost to follow-up.<br><br>In the external cohort, criteria for inclusion were as follows: (a) patients receiving curative surgery for primary NSCLC; (b) the maximum short-axis diameter of N1 and N2 nodes less than 1 cm on CT scan; (c) the SUVmax of N1 and N2 lymph nodes less than 2·5. Criteria for exclusion included (a) multiple lung lesions; (b) poor quality of PET/CT images; (c) patient not receiving SND; (d) patient receiving neoadjuvant therapy; and (e) lost to follow-up.<br><br>In the prospective cohort, criteria for exclusion included (a) multiple lung lesions; (b) poor quality of PET/CT images; (c) participants with incomplete clinical information; (d) participants not receiving SND and (e) participants receiving neoadjuvant therapy.<br><br>In the biopsy cohort, criteria for inclusion were as follows: (a) patients receiving endobronchial ultrasound transbronchial needle aspirations for primary NSCLC; (b) the maximum short-axis diameter of N1 and N2 lymph nodes less than 1 cm on CT scan; and (c) the SUVmax of N1 and N2 lymph nodes less than 2·5. Patients with multiple lung lesions were excluded.
Replication	All codes would be public at Github for replicating our results.
Randomization	The main aim of this study is to evaluate the diagnostic efficiency of DLNMS and most patients are retrospectively collected. Therefore, this study is a modeling study and randomization was applicable.
Blinding	The main aim of this study is to evaluate the diagnostic efficiency of DLNMS and most patients are retrospectively collected. Therefore, this study is a modeling study and blinding was applicable.

Reporting for specific materials, systems and methods

We require information from authors about some types of materials, experimental systems and methods used in many studies. Here, indicate whether each material, system or method listed is relevant to your study. If you are not sure if a list item applies to your research, read the appropriate section before selecting a response.

Materials & experimental systems

n/a	Involved in the study	
☒	☐	Antibodies
☒	☐	Eukaryotic cell lines
☒	☐	Palaeontology and archaeology
☒	☐	Animals and other organisms
☐	☒	Clinical data
☒	☐	Dual use research of concern

Methods

n/a	Involved in the study	
☒	☐	ChIP-seq
☒	☐	Flow cytometry
☒	☐	MRI-based neuroimaging

Clinical data

Policy information about clinical studies

All manuscripts should comply with the ICMJEguidelines for publication of clinical research and a completedCONSORT checklist must be included with all submissions.

Clinical trial registration	NCT05425134
Study protocol	The study protocol for collaection fo the external validation cohort can be assessed at https://clinicaltrials.gov/ct2/show/NCT05425134.
Data collection	In the internal cohort, 1911 patients receiving curative resection for clinical stage N0 non-small cell lung cancer (NSCLC) from January 2018 to December 2019 at Shanghai Pulmonary Hospital were retrospectively reviewed. Criteria for inclusion were as follows: (a) patients receiving curative surgery for primary NSCLC; (b) the maximum short-axis diameter of N1 and N2 lymph nodes less than 1 cm on CT scan; (c) the maximum standardized uptake value (SUVmax) of N1 and N2 lymph nodes less than 2·5. Criteria for exclusion included (a) multiple lung lesions; (b) poor quality of positron emission tomography/computed tomography (PET/CT) images; (c) patient not receiving systematic nodal dissection (SND); (d) patient receiving neoadjuvant therapy; and (e) lost to follow-up.<br><br>In the external cohort, we retrospectively included 355 patients receiving curative resection for clinical stage N0 NSCLC from January 2018 to December 2019 at The First Affiliated Hospital of Nanchang University, Affiliated Hospital of Zunyi Medical College and Ningbo HwaMei Hospital. Criteria for inclusion were as follows: (a) patients receiving curative surgery for primary NSCLC; (b) the maximum short-axis diameter of N1 and N2 nodes less than 1 cm on CT scan; (c) the SUVmax of N1 and N2 lymph nodes less than 2·5. Criteria for exclusion included (a) multiple lung lesions; (b) poor quality of PET/CT images; (c) patient not receiving SND; (d) patient receiving neoadjuvant therapy; and (e) lost to follow-up.<br><br>In the prospective cohort, 999 participants receiving curative resection for clinical stage N0 NSCLC from January 2022 to December 2022 at Shanghai Pulmonary Hospital, The First Affiliated Hospital of Nanchang University, Affiliated Hospital of Zunyi Medical College and Ningbo HwaMei Hospital were enrolled. Criteria for enrollment were as follows: (a) participants scheduled for surgery for radiological finding of pulmonary lesions from the preoperative thin-section CT scans; (b) the maximum short-axis diameter of N1 and N2 lymph nodes less than 1 cm on CT scan; (c) the SUVmax of N1 and N2 lymph nodes less than 2·5; (d) pathological confirmation of primary NSCLC; (e) age ranging from 20-75 years; and (f) obtained written informed consent. Criteria for exclusion included (a) multiple lung lesions; (b) poor quality of PET/CT images; (c) participants with incomplete clinical information; (d) participants not receiving SND and (e) participants receiving neoadjuvant therapy.<br><br>In the biopsy cohort, 366 patients receiving nodal biopsy for clinical stage N0 NSCLC from January 2020 to December 2021 at Shanghai Pulmonary Hospital, The First Affiliated Hospital of Nanchang University, Affiliated Hospital of Zunyi Medical College and Ningbo HwaMei Hospital were retrospective reviewed. Criteria for inclusion were as follows: (a) patients receiving endobronchial ultrasound transbronchial needle aspirations for primary NSCLC; (b) the maximum short-axis diameter of N1 and N2 lymph nodes less than 1 cm on CT scan; and (c) the SUVmax of N1 and N2 lymph nodes less than 2·5. Patients with multiple lung lesions were excluded.
Outcomes	The lymph node staging is used as the primary outcome. The pathologic nodal status in the internal cohort, external cohort and prospective cohort was defined based on surgically resected specimens and that in the nodal biopsy cohort was defined based on biopsy specimens. Survival outcomes are used as the second outcome. Recurrence free survival was defined as the time from the date of treatment to recurrence or death or last follow-up. Overall survival was defined as the time from the date of treatment to death or last follow-up.
